# Novel Butein Derivatives Repress DDX3 Expression by Inhibiting PI3K/AKT Signaling Pathway in MCF-7 and MDA-MB-231 Cell Lines

**DOI:** 10.3389/fonc.2021.712824

**Published:** 2021-08-18

**Authors:** Shailima Rampogu, Seong Min Kim, Baji Shaik, Gihwan Lee, Ju Hyun Kim, Gon Sup Kim, Keun Woo Lee, Myeong Ok Kim

**Affiliations:** ^1^Division of Life Sciences, Division of Applied Life Science (BK21 Plus), Research Institute of Natural Science (RINS), Gyeongsang National University (GNU), Jinju, South Korea; ^2^Division of Life Science and Applied Life Science (BK 21 Plus), College of Natural Sciences, Gyeongsang National University, Jinju, South Korea; ^3^Research Institute of Life Science and College of Veterinary Medicine, Gyeongsang National University, Jinju, South Korea; ^4^Department of Chemistry (BK 21 Plus), Research Institute of Natural Science (RINS), Gyeongsang National University, Jinju, South Korea

**Keywords:** anticancer agents, butein, DDX3, cell cycle, apoptosis

## Abstract

**Background:**

Breast cancer is one of the major causes of mortalities noticed in women globally. DDX3 has emerged as a potent target for several cancers, including breast cancer to which currently there are no reported or approved drugs.

**Methods:**

To find effective cancer therapeutics, three compounds were computationally designed tweaking the structure of natural compound butein. These compounds were synthesized and evaluated for their anticancer property in MCF-7 and MDA-MB-231 cell lines targeting DDX3. The *in silico* molecular docking studies have shown that the compounds have occupied the binding site of the human DDX3 target. Furthermore, to investigate the cell viability effect of **3a**, **3b**, and **3c** on MCF-7 and MDA-MB-231 cell lines, the cell lines were treated with different concentrations of compounds for 24 and 48 h and measured using MTT assay.

**Results:**

The cell viability results showed that the have induced dose dependent suppression of DDX3 expression. Additionally, **3b** and **3c** have reduced the expression of DDX3 in MCF-7 and MDA-MD-231 cell lines. **3b** or **3c** treated cell lines increased apoptotic protein expression. Both the compounds have induced the apoptotic cell death by elevated levels of cleaved PARP and cleaved caspase 3 and repression of the anti-apoptosis protein BCL-xL. Additionally, they have demonstrated the G2/M phase cell cycle arrest in both the cell lines. Additionally, **3c** decreased PI3K and AKT levels.

**Conclusions:**

Our results shed light on the anticancer ability of the designed compounds. These compounds can be employed as chemical spaces to design new prospective drug candidates. Additionally, our computational method can be adapted to design new chemical scaffolds as plausible inhibitors.

## Introduction

RNA helicases are a group of proteins possessing a unique motif, called the DEAD/H (Asp-Glu-Ala-Asp/His) (1). One of the members of the RNA helicase family, DDX3, demonstrates a wide range of roles in the process of cellular biogenesis that includes cell survival, cell-cycle regulation, cellular differentiation, and apoptosis (2). The genome of human encodes for two genes, namely DDX3X and DDX3Y. DDX3Y is present on Y-chromosome and demonstrates its role in male fertility. The gene DDX3X is present on the X-chromosome bands, p11.3– > p11.23 (2) and acts as a oncogene or tumor suppressor (1). DDX3 modulates the expression of genes at various levels. It takes part in transcriptional regulation of gene promoters, engages in splicing, performs the nuclear export of RNA, demonstrates a role in translational regulation (3). The structure of DDX3 is made up of 12 conserved motifs and two recA like domains. The X-ray structure of DDX3 was cocrystallized with the AMP located at the nucleotide-binding pocket (2). The residues from the Q motif, Arg202, and Gln207, hold the adenine group and the phosphate group is held by the P-loop residues, namely Gly227, Ser228, Gly229, Lys230, and Thr231 (2). This binding site has been exploited widely by the researchers to design and discover new chemical compounds (4–6).

DDX3 is a well-studied protein and is a known validated target to design and develop antiviral and anticancer drugs (7). DDX3 communicates with several viral and human proteins and their complexes *via* the RNA and demonstrates a double role in viral replication. Intriguingly, it works as a cofactor for viral replication and acts as a mediator of the innate immunity system (7). The association of DDX3 in cancer has been emerging in the recent times (8) and has demonstrated a significant role in progression of malignancies besides playing a key role in tumorigenesis to metastasis (8–17). Although the role of DDX3 is noticed in several cancers (1), in the current study, we focused on breast cancer targeting two cell lines, MCF-7 and MDA-MB-231, respectively.

DDX3 demonstrates an oncogenic role in breast cancer and upon its elevation may promote cell growth and proliferation (1, 10) and corresponds to distant metastasis (18), whose knockdown represses the tumor volume in vivo (19), thus demonstrating an oncogenic role in breast cancer (1). Several inhibitors were employed to target DDX3 in breast cancer. The combination of DDX3 and PARP inhibitors have brought about synthetic lethality noticed in BRCA1-proficient breast cancer (20). Another compound RK-33 has induced radiosensitization in breast cancer via the mitochondrial translation inhibition (21).

In the current investigation, we have used the butein derivatives to potentially target DDX3 in MCF-7 and MDA-MB-231 cell lines. Butein, a flavonoid, is known to have immense therapeutic potential (22), besides being an anticancer agent (23). In lung cancer cells, butein promotes apoptosis and represses the expression of cyclooxygenase-2 (24). It is reported that butein triggers the suppression of breast cancer growth by causing the reduction of the reactive oxygen species (ROS) production (25) and hindering the AKT phosphorylation. Furthermore, it was mechanistically noticed that butein elicits apoptosis by a series of mechanisms, thereby demonstrating the anti-proliferative activity (26) and can further act as aromatase inhibitor with the IC_50_ value of 3.7 μM (27). It also subsides the proliferative ability of the breast cancer cells by the formation of reactive oxygen species and regulation of ERK and p38 functions (26). Estrogen has been linked with the initiation and the progression of the disease. It was evidenced that butein could efficiently inhibit the aromatase when tested on MCF-7 cells with a minimum inhibitory concentration showing less than 5 µM (27). The purified phenolic-rich EtOAC fraction (PPEF) extracts of medicinal plant Rhus verniciflua Stokes (RVS) that contained butein, demonstrated apoptosis in breast cancer cell lines (28). Fibroblasts, the cockroaches of the human body (29), are found at all the stages of cancer, further playing a major role in promoting the growth of breast cancer. The phytochemical butein has inhibited the clonogenic growth of UACC-812, which was co-cultured with fibroblasts. Mechanistically, it can be speculated that the chalcone butein compound might intrude between the fibroblast and the breast cancer cells at as low as 2.5 µg/ml (30). It is evidenced that butein downregulates the CXC chemo receptor-4 (CXCR4) by transcriptional regulation demonstrated by the downregulation of mRNA expression, suppression of NF-κB activation, and suppression of chromatin immunoprecipitation activity, thereby hindering the process of metastasis (31). It was reported that butein repressed the formation of osteoclasts prompted by MDA-MB-231 cell lines (32).

Motivated by the aforementioned reports, in the current investigation, we have synthesized butein analogs and have tested them against MCF-7 and MDA-MB-231 cell lines, respectively. The primary objective of the study is to evaluate the anticancer potential of the new compounds and to assess their ability as DDX3 inhibitors.

## Methods

### Preparation of the Parent Structure Butein

The butein structure was downloaded from the PubChem database (33) in the 2D format and was subsequently exported on to the *Discovery Studio v18* (hereinafter referred to as DS). The compound was minimized by employing the *Full Minimization* protocol accessible with DS.

### Computational Designing of the Butein Compounds

**Figure 1:**
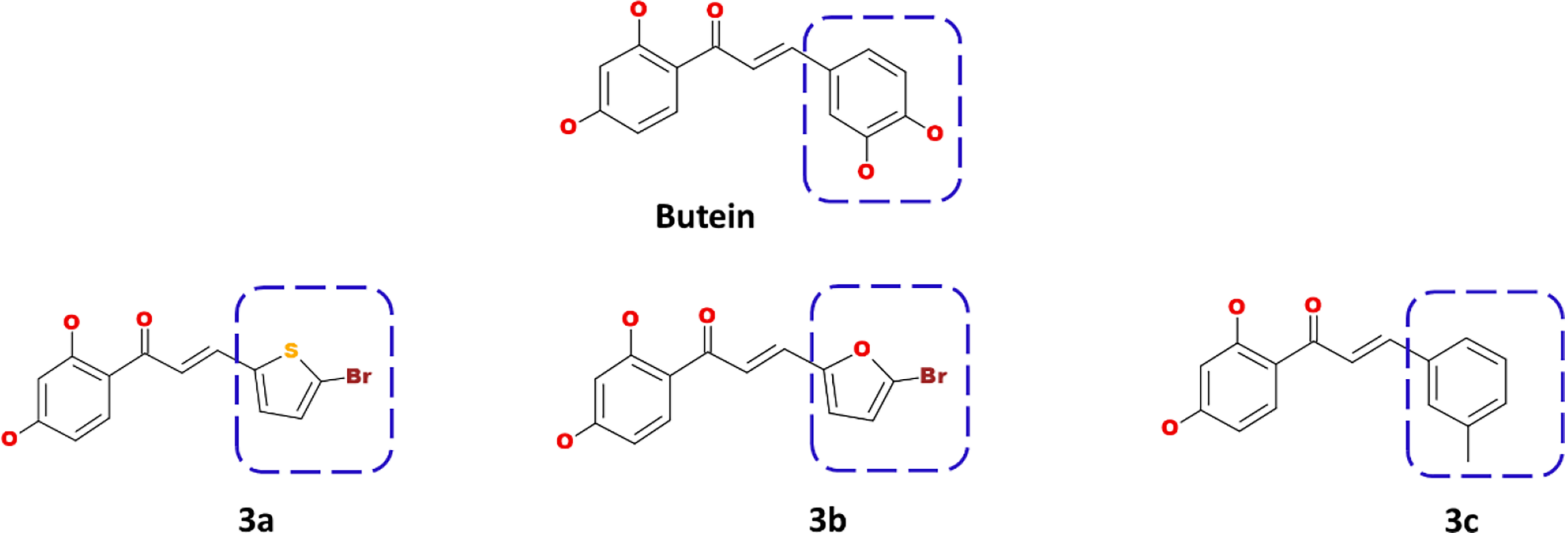
Computational designing of butein compounds modifying the ring B as indicated in dotted box.

Using the *Discovery Studio*, the B ring of butein was modified to obtain new structures as described in **Figure 1**, and three compounds were designed and synthesized.

### Binding Affinity Studies

To elucidate on their binding affinity toward the target DDX3, the molecular docking was performed using the CDOCKER program (34–37) available on DS. The CDOCKER utilized a CHARMm-based molecular dynamics (MD) strategy to dock ligands into a receptor-binding site. Correspondingly, *Random Ligand Conformations* are generated using high-temperature MD. These conformations are then translated into the binding site. The candidate poses are subsequently created using random rigid-body rotations followed by simulated annealing. A final minimization is then used to refine the ligand poses (34, 38).

The protein for the current study is the human DEAD-box RNA helicase DDX3X bearing the PDB code 2I4I cocrystallized with the AMP (39). The protein was initially prepared by initiating the *Clean Protein* tool available with the DS. Correspondingly, the heteroatoms and the water molecules were dislodged and were minimized using the *Minimization* tool available under the *Minimize and Refine Protein* protocol. The active site was marked for all the atoms and the residues around the cocrystallized AMP (39).

The synthesized butein-like compounds were docked into the active site of the protein permitting the generation of 50 conformers for each compound along with the butein compound. The best pose was selected from the largest cluster demonstrating a higher dock score, read according to the -CDOCKER interaction energy and the key residue interactions.

## Synthesis

Unless otherwise noted, all reactions were carried out under an atmosphere of argon in oven-dried flasks. All reagents and chemicals were purchased from Sigma Aldrich Co., TCI, or Alfa Aesar Co. Solvents, such as tetrahydrofuran (THF) and methylene chloride, were used after distillation following standard purification procedures. ^1^H NMR spectra were recorded on a Bruker DRX-300 and chemical shifts (δ) for ^1^H NMR spectra are given in ppm relative to TMS. Column chromatography was performed on Merck silica gel (40–63 mesh).

### Synthesis of 1-(2,4-bis((tert-butyldimethylsilyl)oxy)phenyl)ethanone (2)

To a solution of 2′,4′-dihydroxyacetophenone (0.76 g, 5.0 mmol) in dry CH_2_Cl_2_ (5 mL) triethylamine (2.4 mL, 17.5 mmol) and 4-dimethylamino pyridine (0.06g, 0.5 mmol) were added, and then tertbutyldimethylsilyl chloride (2.11g, 14.0 mmol) dissolved in CH_2_Cl_2_ (9 mL) was added dropwise. After the completion of addition, the reaction mixture was allowed to stirred overnight at room temperature. The reaction mixture was quenched by the addition of water. The organic fractions were collected, and the aqueous phase was extracted with CH_2_Cl_2_. The combined organic fractions were washed with water and brine, dried over anhydrous MgSO_4_, and concentrated under reduced pressure. The residue was purified by short column chromatography on silica gel using n-hexanes as the eluent to afford compound **2** (1.56 g, 82% yield) as a colorless oil; ^1^H NMR (300 MHz, Chloroform-*d*) δ 7.62 (d, *J* = 8.6 Hz, 1H), 6.47 (dd, *J* = 8.6, 2.3 Hz, 1H), 6.32 (d, *J* = 2.3 Hz, 1H), 2.57 (s, 3H), 1.00 (s, 9H), 0.97 (s, 9H), 0.28 (s, 6H), 0.21 (s, 6H).

### Synthesis of Chalcones (3a-3c)

To a solution of compound **2** (1.0 mmol) in dry THF (3 ml) under argon was added lithium diisopropylamide (LDA, 1.0 M, 1.1 ml, 1.1 mmol) dropwise at −78°C. The reaction mixture was allowed to warm to −20°C over 30 min, and then cooled to −78°C. The solution of substituted aldehyde (1.0 mmol) in dry THF (3 ml) was added dropwise at −78°C, and the resulting reaction mixture was allowed to warm to room temperature stirred overnight and then quenched by addition of sat. aqueous NH_4_Cl. The organic fraction was collected, and the aqueous layer was extracted with diethyl ether. The combined organic fractions were washed with brine, dried over anhydrous MgSO_4_, and evaporated under reduced pressure to afford the monohydroxy protected chalcones. The monohydroxy protected chalcones were purified by using CH_2_Cl_2_/MeOH as eluent.

The monohydroxy protected chalcone derivatives (0.2 mmol) were dissolved in THF (5 ml), and tetrabutylammonium fluoride (TBAF, 1.0 M, 0.4 mmol) was added. The resulting reaction mixture was stirred at room temperature for 30 min. and quenched by addition of water. The organic fraction was collected, and the aqueous layer was extracted with ethyl acetate three times. The combined organic fractions were washed with brine, dried over anhydrous MgSO_4_, and evaporated under reduced pressure. The residues were purified column chromatography on silica gel using CH_2_Cl_2_/MeOH as eluent to afford compounds **3a-3c** in 70% to 85% yields.

 
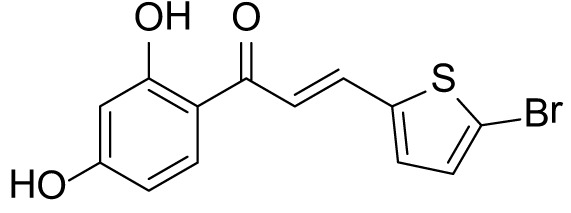


### (*E*)-3-(5-bromothiophen-2-yl)-1-(2,4-dihydroxyphenyl)prop-2-en-1-one (3a)

The compound (**3a**) was synthesized by following the general procedure using monohydroxy-protected chalcone (0.10 g, 0.23 mmol) dissolved in THF (8 mL) and tetrabutylammonium fluoride (TBAF, 1.0 M, 0.46 mL, 0.46 mmol). The **3a** was purified using silica gel column CH_2_Cl_2_/MeOH 10: 1, v/v. Rf. Value = 0.5. Yellow solid (63 mg, 85%); ^1^H NMR (300 MHz, Chloroform-*d*) δ 13.28 (s, 1H), 7.87 (dd, *J* = 15.1, 0.6 Hz, 1H), 7.75 (d, *J* = 8.5 Hz, 1H), 7.23 (d, *J* = 15.1 Hz, 1H), 7.12 (d, *J* = 3.9 Hz, 1H), 7.07 (d, *J* = 3.9 Hz, 1H), 6.46–6.40 (m, 2H), 5.53 (s, 1H).

 
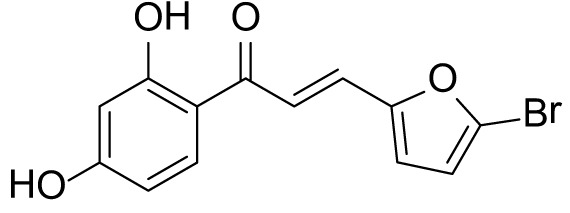


### (*E*)-3-(5-bromofuran-2-yl)-1-(2,4-dihydroxyphenyl)prop-2-en-1-one (3b)

The compound (**3b**) was synthesized by following general procedure using monohydroxy-protected chalcone (0.10 g, 0.24 mmol) dissolved in THF (8 mL) and tetrabutylammonium fluoride (TBAF, 1.0 M, 0.48 mL, 0.48 mmol). The **3b** was purified using silica gel column CH_2_Cl_2_/MeOH 10: 1, v/v. Rf. Value =0.5. Yellow solid (52 mg, 70%); ^1^H NMR (300 MHz, Chloroform-*d*) δ 13.34 (s, 1H), 7.84 (d, *J* = 8.5 Hz, 1H), 7.59–7.38 (m, 2H), 6.67 (d, *J* = 3.5 Hz, 1H), 6.48–6.40 (m, 3H), 5.30 (s, 1H).

 
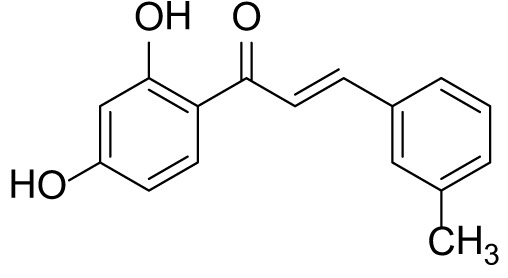


### (*E*)-1-(2,4-dihydroxyphenyl)-3-(m-tolyl)prop-2-en-1-one (3c)

The compound **(3c)** was synthesized by following general procedure using monohydroxy protected chalcone (0.03 g, 0.08 mmol), was dissolved in THF (2 mL) and tetrabutylammonium fluoride (TBAF, 1.0 M, 0.16 mL, 0.16 mmol). The **3c** was purified using silica gel column CH_2_Cl_2_/MeOH 10: 1, v/v. Rf. Value = 0.5. Yellow solid (16 mg, 77%) (40); ^1^H NMR (300 MHz, Chloroform-*d*) δ 13.36 (s, 1H), 7.90–7.83 (m, 2H), 7.56 (d, *J* = 15.5 Hz, 1H), 7.46 (d, *J* = 6.9 Hz, 2H), 7.23–7.35 (m, 2H), 6.47–6.41 (m, 2H), 5.47 (s, 1H), 2.41 (s, 3H); CAS. No. 1385668-91-6.

### Cell Lines and Culture

Human MCF-7 and MDA-MB-231 cell lines were purchased from the Korean Cell Line Bank (KCLB, Seoul, Korea). The MCF-7 and MDA-MB-231 cell lines were maintained in RPMI-1640 medium (Gibco, Life Technologies, Carlsbad, CA, USA) containing 10% (v/v) fetal bovine serum (FBS, Gibco) and 1% penicillin–streptomycin (Gibco) at 37°C in a humidified atmosphere of 5% CO2 (41).

### Cell Viability Assay

The cell viability assay was conducted as described before (42) the cells were seeded in 48 well, plated at a density of 5 × 10^4^ cells in 500 µL medium per well for 16 h. The cultured cells were treated with various concentration for 24, 48, and 72 h. After incubation, the cells were added with 55 µl of 5 mg/ml 3-(4, 5-dimethylthiazol-2-yl)-2, 5-diphenyltetrazolium bromide (MTT; Duchefa Biochemie, Haarlem, The Netherlands) solution for 2 h, and the medium was removed, which was followed by lysis with DMSO. The absorbance at 570 nm was measured with PowerWave HT microplate spectrophotometer (BioTek, Winooski, VT, USA).

### Western Blot Analysis

The Western blot analysis was performed as described before (43). After 48 h treatment of each compounds, the cells were lysed with RIPA buffer (50 mM Tris-HCl pH 7.5, 0.1% SDS, 1% Triton X-100, 150 mM NaCl, 0.5% Sodium deoxycholate, and 2 mM EDTA) containing protease/phosphatase inhibitor cocktail (Thermo Fisher Scientific, Waltham, MA, USA) at 4°C for 1 h. The supernatants were collected and quantified using BCA protein assay kit (Thermo Fisher Scientific) according to the manufacturer’s instructions. To identify the molecular weight, we used Regular Range Protein marker (PM2510; SMOBIO Technology. Inc., Taiwan) and precisionplus protein dual color standard marker (Bio-Rad catalog no 1610374). Protein of 10–20 µg concentration was loaded on 8–12% sodium dodecyl sulfate polyacrylamide gel electrophoresis (SDS-PAGE), and then transferred to polyvinylidene difluoride membrane (PVDF, ATTO, Tokyo, Japan). The membranes were blocked with TBS-T buffer (Tris-buffered saline containing 0.1% Tween 20) containing 5% (w/v) skim milk power for 1 h at 25°C, and then incubated with primary antibodies for 16 h at 4°C. After incubation with the secondary antibody for 3 h at 25°C, the membranes was detected using Clarity™ Western ECL Blotting Substrates (Bio-Rad, Hercules, CA, USA).

### Statistical Analysis

The data were expressed as mean ± standard error of mean (SEM) and analyzed by GraphPad Prism Version 4.0b (GraphPad Software Inc., La Jolla, CA, USA), for statistical significance using one-way analysis of variance (ANOVA). p < 0.05 was considered as statistically significant. All experiments were performed in triplicates.

## Results

### Computational Designing of the Butein-Compounds

The prepared butein compound was used as the starting structure (parent structure) to computationally design new compounds. Subsequently, modifying the ring B has yielded three compounds as shown in **Figure 1**.

### Binding Affinity Studies

The synthesized compounds were assessed for the binding affinity studies using the CDOCKER program. These compounds were docked into the ATP binding pocket of the DDX3 to understand their potentiality along with butein for comparative study. The results have shown that the modified compounds have demonstrated a better to comparable dock score than the parent compound butein as shown in **Table 1**.

**Table 1:** Molecular dock score of butein and its analogues with target DDX3.

Compound name	-CDOCKER Interaction Energy (kcal/mol)
Butein	33.23
3a	34.08
3b	34.07
3c	35.55

**Figure 2:**
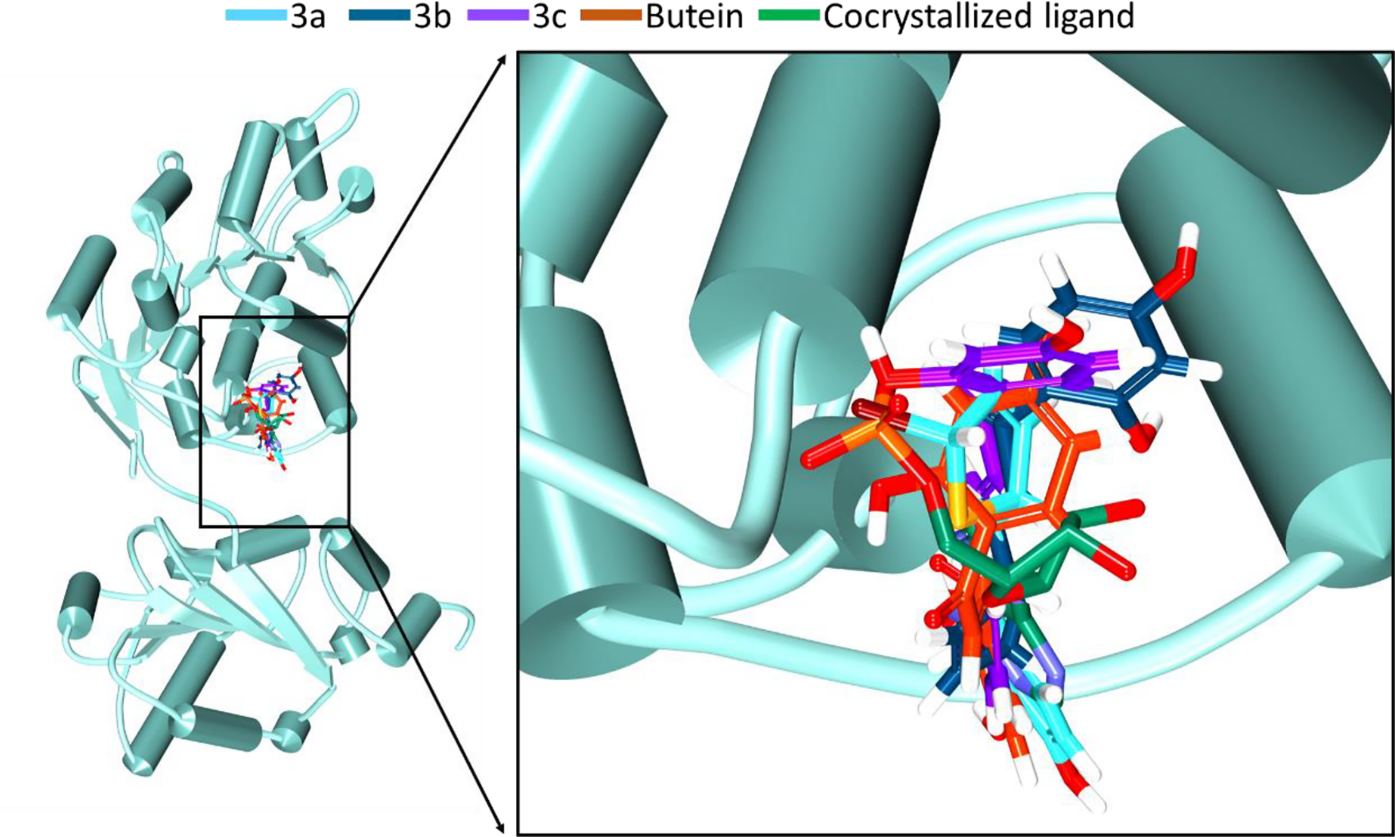
Accommodation of the synthesized compounds at the ATP binding pocket of DDX3 in comparison with butein and the cocrystallized ligand. The new compounds have displayed a similar binding mode as that of the cocrystallized ligand.

**Figure 3:**
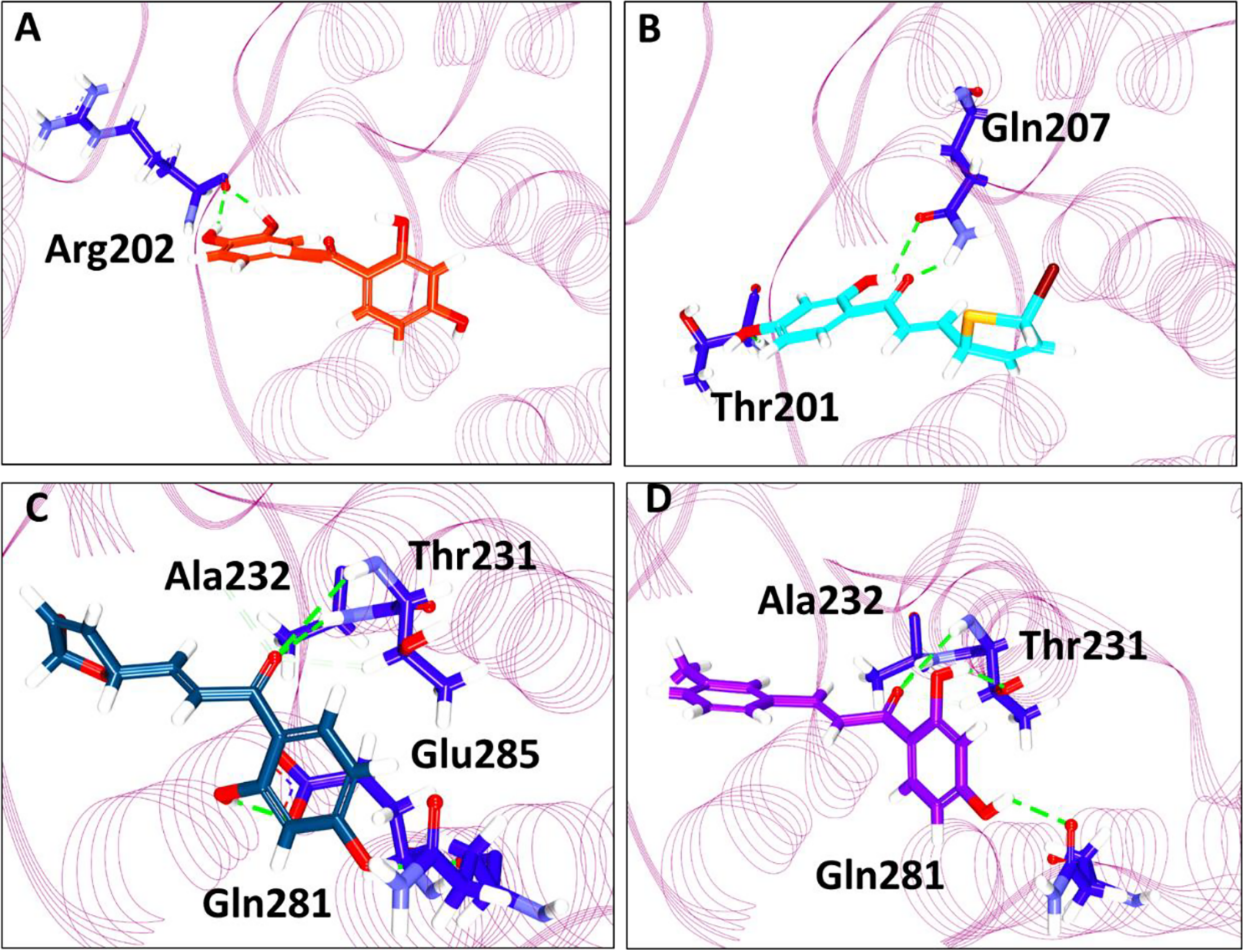
Intermolecular interaction between the protein and the small molecules. **(A)** Interaction of compound butein. **(B)** Hydrogen bond interaction between DDX3 and **3a**. **(C)** Molecular interactions between **3b** and DDX3. **(D)** Hydrogen bond interaction of DDX3 and **3c**.

Further, the compounds have shown interactions with the key residues positioning at the binding pocket as elucidated in **Figure 2**. The compound butein has formed hydrogen bond with the residue Arg202, as displayed in **Figure 3A**. Additionally, the key residue Tyr200 has formed the π-π stacked interaction. The **3a** has prompted hydrogen bond interactions with the residues, Thr201 and Gln207, as shown in **Figure 3B**, along with the π-π stacked interaction by Tyr200. The compound **3b** has interacted *via* hydrogen bonds with the residues, Thr231, Ala232, Gln281, and Glu285, respectively, as represented in **Figure 3C**. The key residue Tyr200 has participated by π-alkyl interaction holding the compound at the binding pocket. The compound **3c** has formed hydrogen bond interactions with the residues, Thr231, Ala232, and Gln281 residues. The residue Thr231 has formed two hydrogen bonds with the ligand as illustrated in **Figure 3D**. The key residue Tyr200 has demonstrated the π-π stacked interaction. Several other residues have clamped the ligands at the binding pocket as shown in **Supplementary 1A–C** respectively.

### Synthesis of Chalcones

**Scheme 1:**
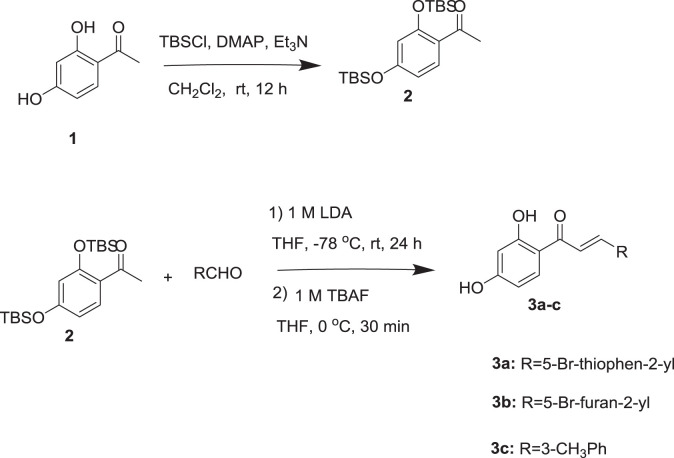
Synthesis of chalcones.

The syntheses of chalcones are illustrated in **Scheme 1**. All the chalcones were synthesized by aldol condensation reaction using an appropriate aldehyde and ketone as previously described in the literature method with modification. The dihydroxy ketone was protected by using TBSCl. Dihydroxy protected compound by reacting with aldehyde in the presence of LDA base resulted in the condensation product, which on desilylation by TBAF, the desired chalcones were obtained. The ^1^H NMR spectrum data of the three compounds is provided as, **Supplementary Figures 2–4** for **3a**, **3b**, and **3c**, respectively.

### Anti-Proliferative Effect of 3b and 3c

**Figure 4:**
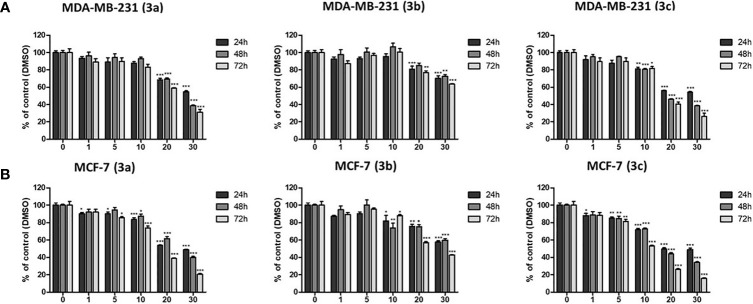
The cytotoxic effects on **3a**, **3b**, and **3c** in MDA-MB-231, and MCF-7 cell lines. **(A)** The MDA-MB-231 and **(B)** MCF-7 cells were treated with each compounds as various concentrations (0–30 μM) for 24, 48, and 72 h. Then, the cell viability was measured by MTT assay. *p < 0.05 *vs*. untreated group; **p < 0.01 *vs*. untreated group; ***p < 0.001 *vs*. untreated group.

To evaluate the anti-proliferative effect of **3b** and **3c**, MTT assays were carried out on the MDA-MB-231 and MCF-7 representing the human breast cancer cell lines. **3b** and **3c** were treated with various concentration for 24, 48, and 72 h in the MDA-MB-231 and MCF-7, respectively. The cell viability on the MDA-MB-231 and MCF-7 cells was markedly decreased by **3b** and **3c** treatment in a dose- and time-dependent manner as shown in **Figures 4A, B**. The IC_50_ values of **3b** were recorded as 58.23 μM and 37.74 μM at 48 h in MCF-7 and MDA-MB-231 cell line, respectively. Also, IC_50_ values of **3c** were shown as 22.72 μM and 20.51 μM at 48 h in MCF-7 and MDA-MB-231 cell line, respectively. These results showed that **3b** and **3c** have anti-proliferative effect in both human cell lines. Therefore, we used 20 and 30 μM of **3a**, 25 and 50 μM of **3b**, and 10 and 20 μM of **3c** in our subsequent experiments.

### Inhibition Effect of DDX3 Protein Level on 3b and 3c

**Figure 5:**
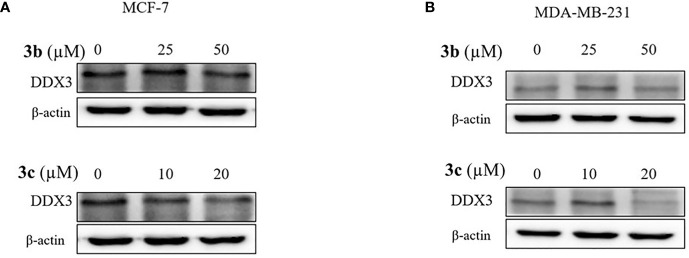
DDX3 protein expression on **3b** and **3c** in MCF-7 and MDA-MB-231 cell lines. The **3b** and **3c** were treated with indicated concentration **(A)** MCF-7 **(B)** MDA-MB-231 cell lines for 48 h. Control group (0 μM) was treated with same amount of DMSO. Western blot analysis were conducted to identify the DDX3 protein expression. The β-actin protein was used as loading control.

Based on cell viability results, we also identified the DDX3 protein expression. The two cell lines, which include MCF-7 and MDA-MB-231 cells were treated with indicated concentration of **3a**, **3b**, or **3c**, respectively. The DDX3 protein expression was decreased dose-dependently in **3b** or **3c** treated MCF-7 and MDA-MB-231 cell lines as shown in **Figures 5A, B**, and **Supplementary Figure 5**. This data indicated that **3b** and **3c** could suppress DDX3 protein expression in MCF-7 and MDA-MB-231 cell lines. Since **3a** did not show any significant reduction of DDX3 expression, as shown in **Supplementary Figure 7**, we have not proceeded with the compound further.

### Apoptosis and Cell Cycle Arrest Effect of 3b and 3c

**Figure 6:**
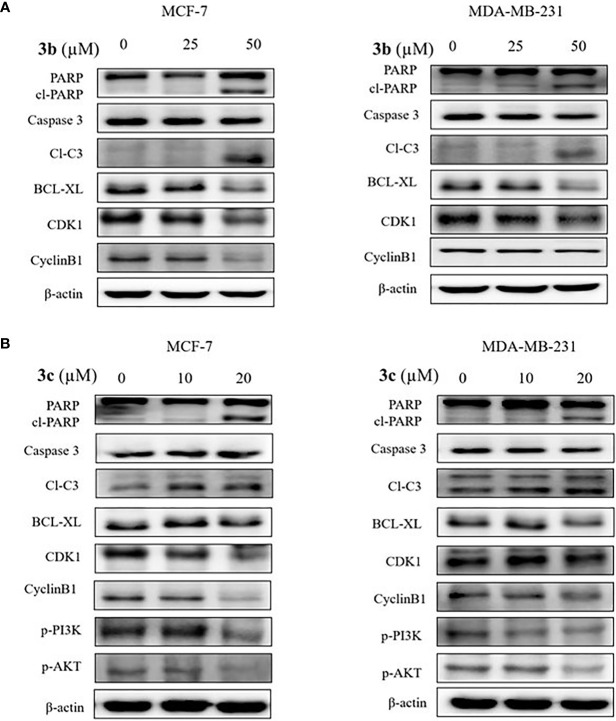
The apoptotic cell death effects of **3b** and **3c** in MCF-7 and MDA-MB-231 cell lines. The indicated concentration of **3b** was treated in the MCF-7 and MDA-MB-231 cell lines for 48 h. **(A)** The indicated concentration of **3c** was treated in the MCF-7 and MDA-MB-231 cell lines for 48 h. **(B)** Control group (0 μM) was treated with same amount of DMSO. The apoptotic related protesins (PARP, cleaved PARP, cleaved caspase 3, BCL-XL), cell cycle related proteins (CDK1 and CyclineB1), p-PI3K and p-AKT expression levels were analyzed using Western blot. The β-actin protein was used as loading control.

To determine the cell death and cell cycle arrest effect on **3b** and **3c** in human breast cancer cell lines, we identified the apoptosis and cell cycle related protein expression in **3b** and **3c** treated MCF-7 and MDA-MB-231 cell lines. The apoptosis marker proteins, such as cleaved PARP and cleaved caspase 3 were increased, and anti-apoptosis protein, BCL-xL, was decreased in compound use to treat both cell lines, as shown **Figures 6A, B**, and **Supplementary Figure 6**. In addition, G2/M cell cycle arrest-related protein including CDK1 and cyclinB1, were inhibited in compound treated both cell lines. Those data suggest that **3b** and **3c** can induce apoptotic cell death and G2/M phase cell cycle arrest in MCF-7 and MDA-MB-231 cell lines, indicating anti-cancer effect, as shown in **Figures 6A, B**.

In addition, PI3K/AKT signaling pathway is an important intercellular signal involving apoptotic cell death and cell cycle arrest. Therefore, we examined the phosphorylated PI3K and AKT protein expression in the **3b** and **3c** treated human breast cancer cells. **3b** was not affective to PI3K/AKT protein change. The results showed that inhibition of PI3K and AKT activation was induced in the **3c** treated both cell lines, as shown in **Figure 6B**.

## Discussion

With an objective to find effective therapeutics to breast cancer, the current investigation has proceeded by tweaking the natural compound butein. Correspondingly, we have obtained three compounds. These three compounds, namely **3a**, **3b**, and **3c**, were docked into the active site of the protein target 2I4I to gain insights into the atomistic interactions. Alluringly, the three compounds have rendered an interaction with the key residue Tyr200, *via* π-π stalk interaction with **3a** and **3c**, whereas **3b** has prompted a π-alkyl interaction as shown in **Supplementary Figure 1**. Interaction with this residue is regarded to be potential as reported in previous reports (2, 4, 44). Additionally, it was reported that binding with Tyr200 phenyl ring and the imidazole-diazepine ring amplifies the binding (19). It was further noted that the compounds were accommodated at the binding pocket prompted by various residues.

To elucidate on their anti-proliferative activity in cancer cells, we proceeded with the *in vitro* MTT assay. This assay has shown a remarkable decline in the cell viability when compared with the untreated cells. The **3a** has shown an IC_50_ value of 27.44 and 26.06 μM in MCF-7 and MDA-MD-231 cell lines for 48 h, whereas the **3b** and **3c** have demonstrated 58.23 μM and 37.74 μM and 22.72 μM and 20.51 μM, respectively, in MCF-7 and MDA-MD-231 cell lines recorded at 48 hrs. Although, **3a** showed relatively stronger IC_50_ value, it has not repressed the DDX3 expression. This gives rise to the notion that **3a** might act on other target other than DDX3, while the **3b** and **3c** have decreased the expression of DDX3 in both the cell lines. This finding sheds light on the identification of new inhibitors against DDX3.

Fundamentally, the flavonoids promote the anticancer mechanism by instigating the apoptosis and cell cycle arrest (42). The apoptosis was studies by the expression of cleaved PARP and cleaved caspase 3 (42). Caspases broadly belong to the protease family that manifests specificity to aspartic acids. These caspases are pivotal players of apoptosis (42). Among the caspases, Caspase-8, -9, and -10 are called the instigators of apoptosis as they are capable of activating other caspases while the caspase-3, -6, and -7 are grouped as executioners because of their ability to cleave important substrates thereby killing the cell (45). One such substrate is the poly (ADP-Ribose) polymerase (PARP) that is instrumental in maintaining DNA stability and repair (45, 46). The PARP being cleaved into two fragments serves as an indication of functional caspase activation (45). Our identified compounds have triggered the elevation of the apoptosis marker protein, such as cleaved PARP and cleaved caspase 3.

The antiapoptosis protein (apoptosis inhibitor) is an eminent member of BCL-2 family of apoptosis regulators (47). This protein is elevated in certain cancers, and its role in particular to breast cancer corresponds to increased metastatic potential than the primary tumor growth (48, 49) and is a target against invasive cancer cells (50). Our finding has shown that the level of BCL-xL was downregulated by both the compounds in two cell lines. These results signify the ability of the compounds to induce apoptosis cell death.

We further extended our study to assess the effect of **3b** and **3c** on cell cycle. Cell cycle has four phases that transvers from quiescence (G0 phase) to proliferation (G1, S, G2, and M phases). The process of cell cycle occurs by the activation of cyclin-dependent kinase (CDK) and its corresponding cofactor the cyclins (51). Among the innumerable factors that are involved in the process, the activation of CDK-cyclin heterodimeric complexes are considered as the paramount steps. Furthermore, the activation of the kinases (CDKs) is closely governed by the binding to cyclins (51). Correspondingly, the cell cycle proteins, cyclin B1 and CDK1, are interconnected with the G2/M phase. The primary factor cyclin B1 switches on the mitosis could compose compound with CDK1 to adjust the G2/M phase (51). Our findings have demonstrated that **3b** and **3c** have arrested the G2/M phase and suppressed the proteins CDK1 and cyclin B1 (51). The corresponding downregulation of CDK1 and cyclin B1 may reduce the formation of CDK1-cyclin B1 complex, thereby leading to the arrest of G2/M phase. These findings put forth that **3b** and **3c** have arrested the G2/M phase by repressing the expression of proteins CDK1 and cyclin B1.

**Figure 7:**
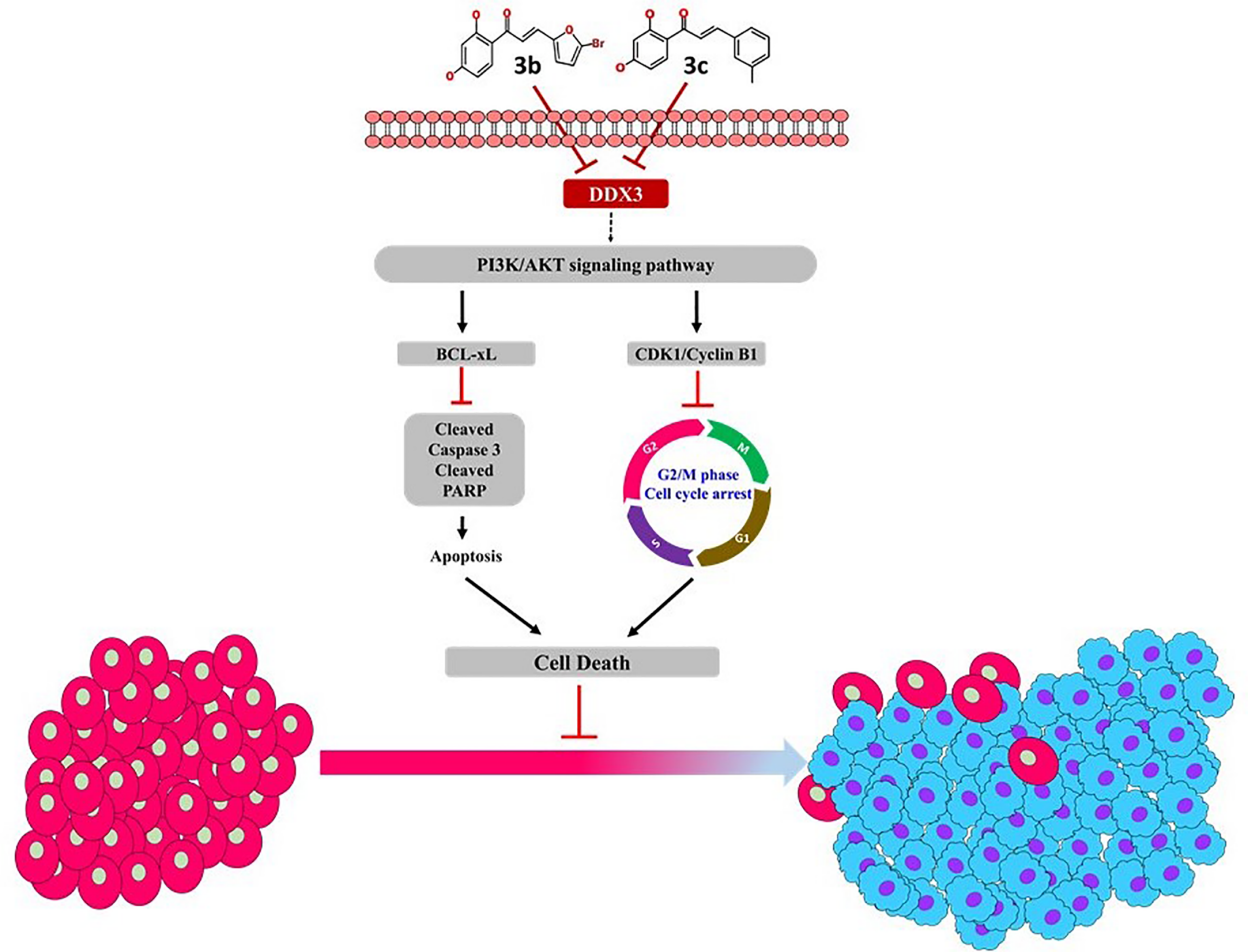
Proposed mechanism of **3b** and **3c**.

The phosphatidylinositol 3-kinase (PI3K)/protein kinase B (AKT) signaling pathway is believed to be associated with the modulation of several cellular physiological processes (52) and has an important role in the regulation of cell proliferation, the cell cycle and apoptosis (52). It is a well-known fact that inhibition of PI3K/AKT signal pathway favors the process of apoptosis in cancer cell (53, 54). Likewise, **3c** inhibited the expression level of PI3K and its downstream target AKT that is remarkably associated with cellular apoptosis. In a noteworthy observation, it was only **3c** that could inhibit the PI3K/AKT signal pathway. The proposed mechanism of inhibition is represented in **Figure 7**, and all the full blots are provided as **Supplementary Files**.

In conclusion, the present manuscript predominantly focuses on finding new therapeutics targeting DDX3. DDX3 is widely noted target that is over expressed in several cancers. In here, the phytocompound butein has been modified at ring B position to obtain new derivatives. These new compounds have repressed DDX3 expression in two cell lines, illuminating their potential to target DDX3. Additionally, these compounds have arrested the cell cycle proteins and have shown apoptosis, remarkably extending their usability in cancer treatments. Taken together, we propose two butein derivatives as novel DDX3 inhibitors that can additionally serve as scaffolds for designing new compounds.

## Data Availability Statement

The original contributions presented in the study are included in the article/**Supplementary Material**. Further inquiries can be directed to the corresponding authors.

## Author Contributions

SR, GL, MK, and KL conceived the idea. SR and GL performed the computational designing and molecular docking. BS and JK have synthesized the compounds. SK and GK have performed the MTT assay and the western blot analysis. SR, BS, and SK wrote the manuscript. All authors contributed to the article and approved the submitted version.

## Funding

This research was supported by the Bio & Medical Technology Development Program of the National Research Foundation (NRF) and funded by the Korean government (MSIT) (No. NRF-2018M3A9A7057263). This research was supported by the Neurological Disorder Research Program of the National Research Foundation (NRF) funded by the Korean Government (MSIT) (2020M3E5D9080660).

## Conflict of Interest

The authors declare that the research was conducted in the absence of any commercial or financial relationships that could be construed as a potential conflict of interest.

## Publisher’s Note

All claims expressed in this article are solely those of the authors and do not necessarily represent those of their affiliated organizations, or those of the publisher, the editors and the reviewers. Any product that may be evaluated in this article, or claim that may be made by its manufacturer, is not guaranteed or endorsed by the publisher.

## Abbreviations

DEAD/H, Asp-Glu-Ala-Asp/His; BRCA1, breast cancer 1; CXCR4, CXC chemo receptor-4; CDK1, cyclin-dependent kinase 1; KCLB, Korean cell line bank; IC50, half-maximal inhibitory concentration; MD, molecular dynamics; PPEF, purified phenolic-rich EtOAC fraction; PARP, poly (ADP-ribose) polymerase; SEM, standard error of mean; TNFα, tumor necrosis factor alpha; THF, tetrahydrofuran; TBAF, tetrabutylammonium fluoride.
