# Supplementary material for: Novel Butein Derivatives Repress DDX3 Expression by Inhibiting PI3K/AKT Signaling Pathway in MCF-7 and MDA-MB-231 Cell Lines
Source: Front Oncol. 2021 Aug 18;11:712824. doi: 10.3389/fonc.2021.712824 (PMC8416463; doi:10.3389/fonc.2021.712824)
Supplement: Supplementary file 1 [file DataSheet_1.pdf]

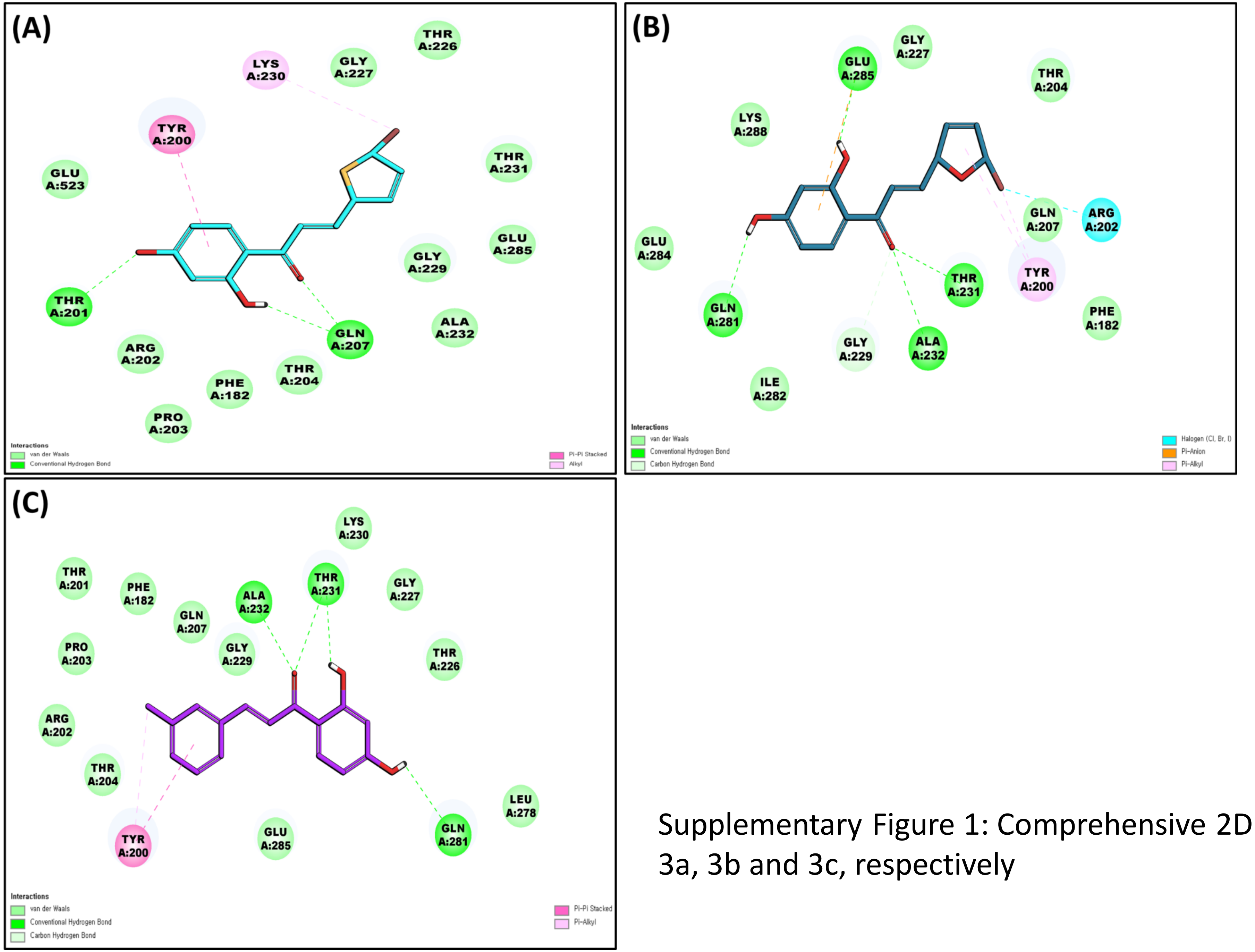

Supplementary Figure 1: Comprehensive 2D interactions of 3a, 3b and 3c, respectively

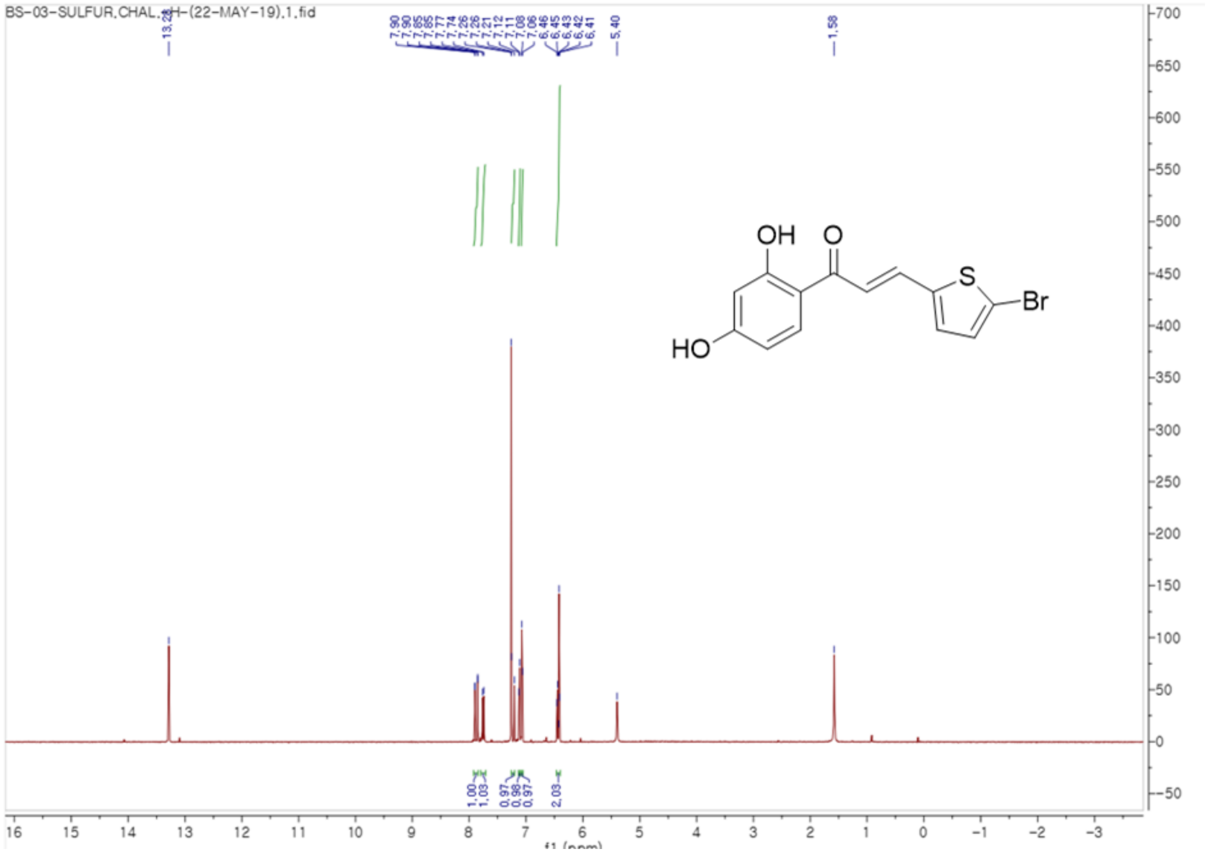

Supplementary Figure 2. ^{1}H NMR spectrum of Compound **3a**

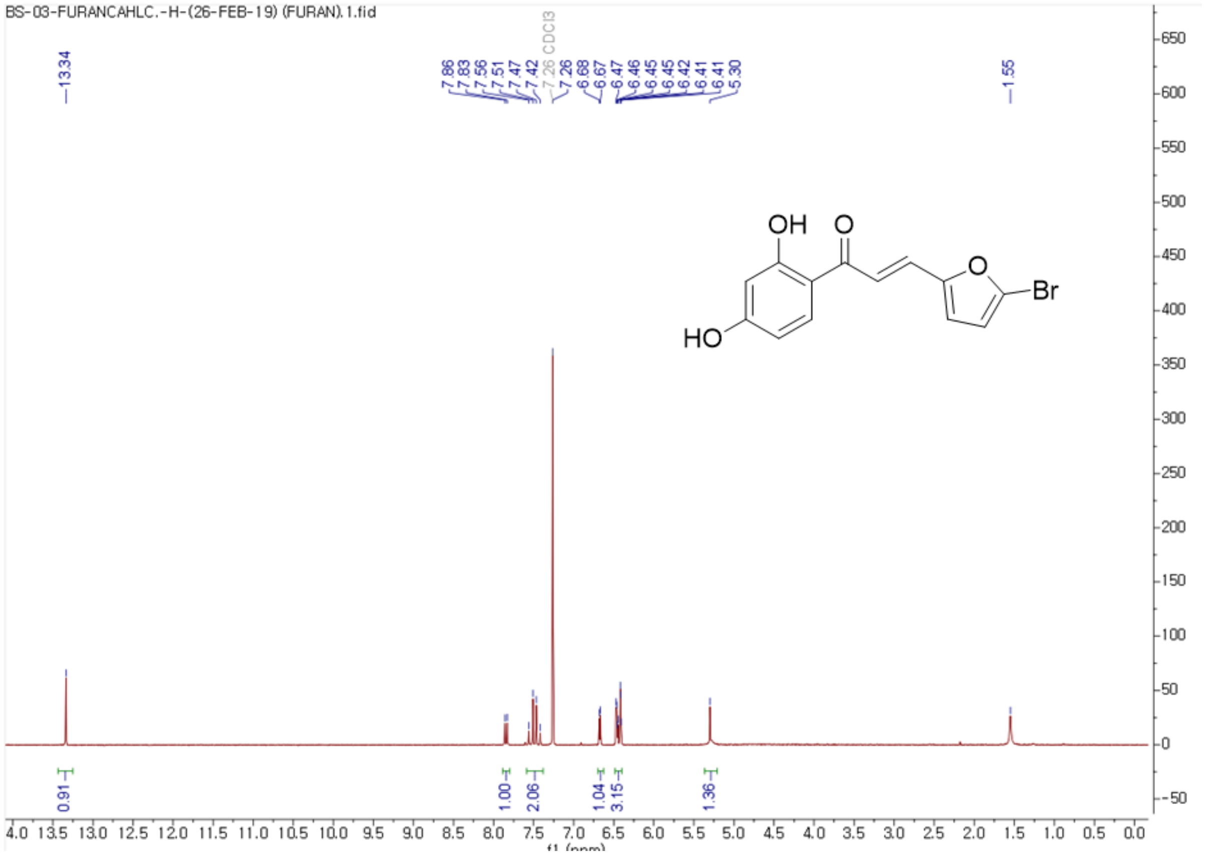

Supplementary Figure 3. ^{1}H NMR spectrum of Compound **3b**

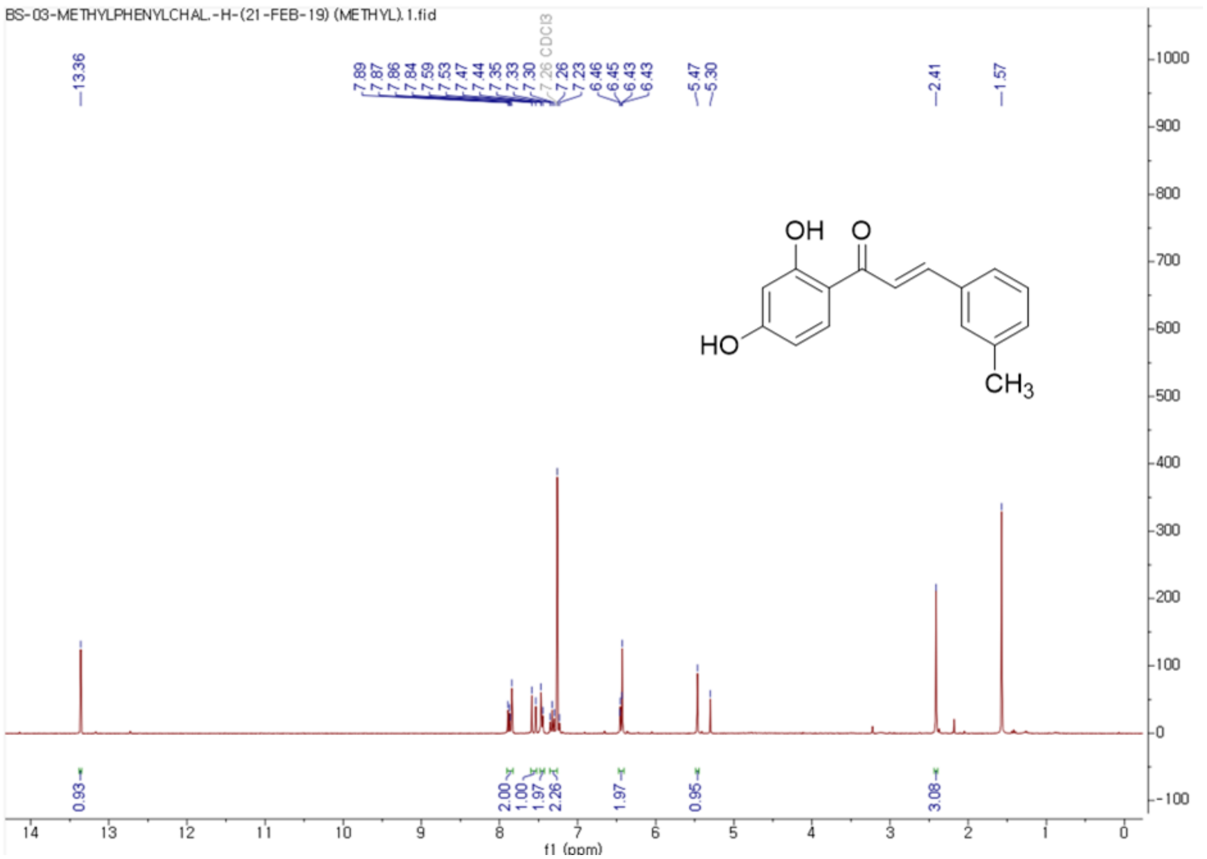

Supplementary Figure 4. ^{1}H NMR spectrum of Compound **3c**

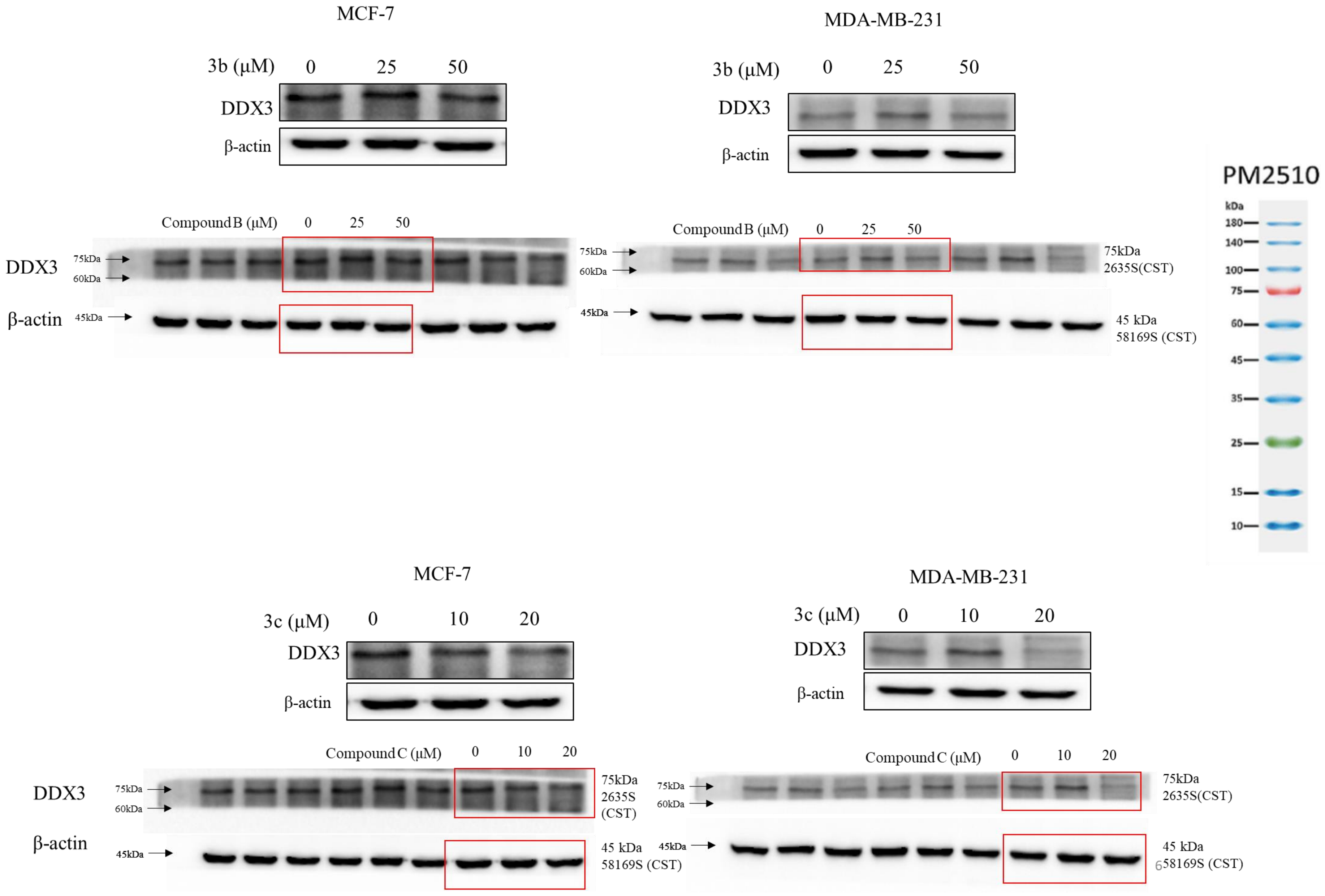

Supplementary Figure 5. Full blots of Figure 5

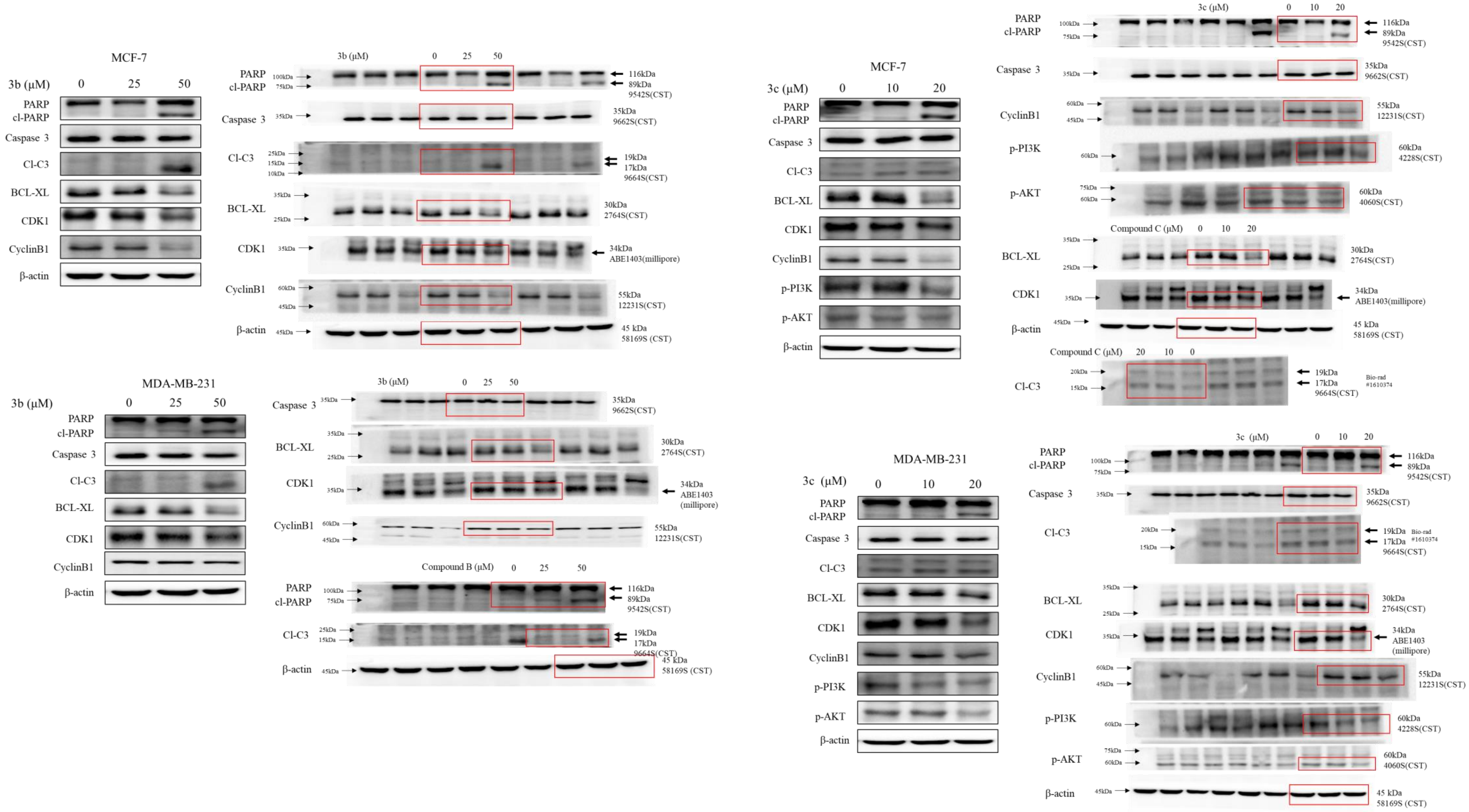

Supplementary Figure 6. Full blots of Figure 6

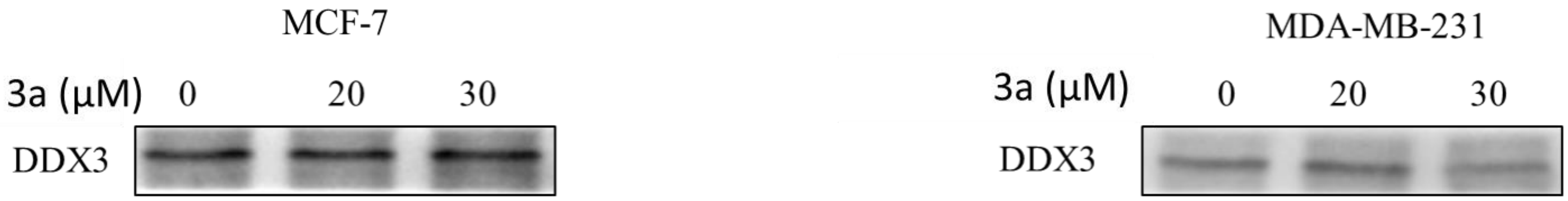

Supplementary Figure 7. DDX3 protein expression on **3a**
